# Euphresco Sendo: An international laboratory comparison study of molecular tests for *Synchytrium endobioticum* detection and identification

**DOI:** 10.1007/s10658-017-1411-6

**Published:** 2018-02-07

**Authors:** Bart van de Vossenberg, Marcel Westenberg, Ian Adams, Olga Afanasenko, Ani Besheva, Margriet Boerma, James Choiseul, Toos Dekker, Kerstin Flath, Marga van Gent-Pelzer, Kurt Heungens, Anatolii Karelov, Ilona Kibildiene, Jaroslaw Przetakiewicz, Alexandra Schlenzig, Vera Yakovleva, Gerard van Leeuwen

**Affiliations:** 1 Dutch National Plant Protection Organization, National Reference Centre, Geertjesweg 15, 6706EA, Wageningen, The Netherlands; 2 Fera Science Ltd., Sand Hutton, York, YO41 1LZ UK; 3 All Russian Research Institute for Plant Protection, Podbelsky sh. 3, Pushkin, Saint Petersburg, Russia; 4 Central laboratory for plant quarantine, 120, N. Moushanov Blvd, 1330 Sofia, Bulgaria; 5 Hilbrands Laboratorium BV, Kampsweg 27, 9418 PD Wijster, The Netherlands; 6 Department of Agriculture, Food and the Marine, Backweston Campus, Celbridge, Co. Kildare Ireland; 7 NAK, Randweg 14, 8304 AS Emmeloord, the Netherlands; 8 Julius Kühn-Institut, Stahnsdorfer Damm 81, 14532 Kleinmachnow, Germany; 9 Wageningen Plant Research, BU Biointeractions & Plant Health, Droevendaalsesteeg 1, 6708 PB Wageningen, The Netherlands; 10 Institute for Agricultural and Fisheries Research, Plant Unit, Burg. Van Gansberghelaan 96, 9820 Merelbeke, Belgium; 11 Institute of Plant Protection, 33 Vasylkivska Str, Kiev, 3022 Ukraine; 12 The State Plant Service, Phytosanitary Research Laboratory (Division), Ministry of Agriculture, Sukileliu str. 9A, LT - 11352 Vilnius, Lithuania; 13 Plant Breeding and Acclimatization Institute, National Research Institute, Radzikow, 05-870 Blonie, Poland; 14 Science and Advice for Scottish Agriculture, 1 Roddinglaw Road, Edinburgh, EH12 9FJ UK; 15 All-Russian Plant Quarantine Center, Pogranichnaya 32, Bykovo, 140150 Ramenskoe region, Moscow, Oblast Russia

**Keywords:** Validation, Test Performance Study, Inter-laboratory comparison study, EPPO Diagnostic Standard, Potato wart disease

## Abstract

An international test performance study (TPS) was organised to generate validation data for three molecular *Synchytrium endobioticum* tests: van den Boogert et al. (*European Journal of Plant Pathology 113,* 47–57, 2005), and van Gent-Pelzer et al. (*European Journal of Plant Pathology, 126,* 129-133, 2010) for the detection of *S. endobioticum*, and the pathotype 1(D1) identification test described by Bonants et al. (*European Journal of Plant Pathology, 143*, 495-506, 2015). Two TPS rounds were organised focussing on different test matrices, i.e. round 1: warted potato tissue, and round 2: resting spore suspensions. When using the tests for detection and identification of *S. endobioticum* in warted potato tissue, no significant differences were observed for diagnostic sensitivity, diagnostic specificity, overall accuracy, analytical sensitivity and robustness. When using the tests for detection and identification of *S. endobioticum* in resting spore suspensions, the van den Boogert and van Gent-Pelzer tests significantly outperform the Bonants test for diagnostic sensitivity and diagnostic specificity. For overall accuracy and analytical sensitivity, the van Gent-Pelzer significantly outperforms the van den Boogert and Bonants tests and is regarded as the test of choice when identifying *S. endobioticum* from resting spores. Tests regarded fit for purpose for routine testing of wart material and resting spore suspensions are proposed for the update of EPPO standard PM7/28(1) *Synchytrium endobioticum*.

**Electronic supplementary material:**

The online version of this article (10.1007/s10658-017-1411-6) contains supplementary material, which is available to authorized users.

## Introduction

The cosmopolitan soil-borne obligate biotrophic fungus *Synchytrium endobioticum* (Schilb.) Perc. is the causal agent of potato wart disease and is considered one of the most important quarantine organisms of cultivated potatoes (Smith *et al*. 1997, Obidiegwu *et al.* 2014). Upon infection, *S. endobioticum* induces tumor-like growth (galls or warts) in host tissues of susceptible potato cultivars resulting in yield losses up to 100% (Hampson 1993). Robust resting spores are formed in the warted tissue and are released into the surrounding soil when host tissue decays. These resting spores, which can remain viable and infectious in soil for decades (Laidlaw 1985; Przetakiewicz 2015a), together with the lack of successful chemical control agents (Hampson 1993), present a major challenge to potato production.

To date, 39 pathotypes of the fungus have been described (Baayen *et al.* 2006, Przetakiewicz 2015b) and phytosanitary measures heavily rely on pathotype identification. The main focus of the current version of the European and Mediterranean Plant Protection Organization (EPPO) *S. endobioticum* standard PM7/28 (EPPO 2004) lies with pathotype identification using different bioassays, and no molecular tests for pathogen detection or identification are included. We aim to fill this gap by generating validation data for three molecular tests, including both DNA extraction and amplification, in an international test performance study (TPS).

Two TPS rounds were organised focussing on different test matrices (i.e. warted potato tissue in round 1, and resting spore suspensions in round 2). TPS results and additionally generated data were used to evaluate the following performance criteria: analytical sensitivity, analytical specificity, diagnostic sensitivity, diagnostic specificity, repeatability and robustness following EPPO standard PM7/98 (EPPO 2014a). A draft version of the EPPO standard on the organisation of interlaboratory comparison studies (EPPO 2014b) was used for guidance during the TPS setup.

Three molecular tests for *S. endobioticum* detection were described in literature when setting up the TPS; a conventional PCR amplifying 543 bp of the internal transcribed spacer (ITS) region (Niepold and Stachewicz 2004); a conventional PCR amplifying 472 bp of the ITS region (van den Boogert *et al.* 2005); and a real-time PCR TaqMan test amplifying 84 bp of the internal transcribed spacer 2 (ITS2) (van Gent-Pelzer *et al.* 2010). A short informal questionnaire held among laboratories working with *S. endobioticum* indicated that tests described by van den Boogert *et al*. and van Gent-Pelzer *et al*. were most frequently used. For that reason both tests were included in this study together with a recently developed real-time PCR test for *S. endobioticum* pathotype 1(D1) identification (Bonants *et al.* 2015), which was unpublished at the time of TPS preparation. The latter test, targeting a pathotype 1(D1) associated single nucleotide polymorphism (SNP), is the first example of molecular *S. endobioticum* pathotype identification using real-time PCR and consists of a duplex test targeting pathotype 1(D1) and non-1(D1) pathotypes. In addition to the specific tests, a TaqMan test targeting the plant COI gene was used as an internal control (Mumford *et al.* 2004). In this paper, tests described by van den Boogert *et al.*, van Gent-Pelzer *et al.*, Bonants *et al.* and Mumford *et al*. are referred to as Sendo PCR, Sendo TaqMan, 1(D1) and non-1(D1) TaqMan, and COX TaqMan respectively.

Tests regarded as fit for purpose based on the performance criteria determined in this study were suggested for addition to the update of PM7/28.

## Materials and Methods

### Participants

Fifteen laboratories involved in research or diagnostic activities for *S. endobioticum* with at least two years of experience with molecular techniques participated in the study. A workshop with training session was organised before the start of the first TPS round to familiarise participants with the TPS setup and the tests included.

### Sample set preparation

Warted potato tissue was used as starting material for DNA extraction in the first TPS round. The sample set consisted of randomised healthy potato pieces and wart pieces taken from *S. endobioticum* pathotypes 1(D1), 2(G1), 6(O1), 18(T1), and 38(Nevsehir) infected potatoes. Cuttings of warted tissue from a single isolate was used to prepare several TPS samples. For instance 2(G1) warts (MB08) were used to prepare sample 1, sample 9 and the return sample in TPS round 1 (Table 1). Healthy potatoes of cv. “Eersteling” and warts were cut in portions of approximately 100 mg, added to 2 mL lyophilisation ampoules (VWR, Radnor, USA) and frozen 16 h at −80 °C prior to lyophilisation with a BenchTop 4 K BTXL-75 freeze-dryer (VirTis, Warminster, USA). Ampoules were closed under vacuum and topped off with a tear-away crimp cap (VWR, Radnor, USA).

**Table 1:** TPS samples from round 1 (wart material) and round 2 (resting spores), and their assigned qualitative values based on homogeneity test results

TPS round	Control/sample	Material	pathotype/ cultivar	Strain	Assigned evalues		
					Sendo PCR	Sendo TaqMan	1(D1) TaqMan
1 & 2	NAC	MGW^a^	–	–	–	–	–
1 & 2	PAC1 1(D1)	Wart DNA	1(D1)	MB42	+	+	1(D1)
1 & 2	PAC2 1(D1)	10^−2^ PAC1 1(D1)	1(D1)	MB42	+	+	1(D1)
1 & 2	PAC1 non-1(D1)	Wart DNA	2(G1)	MB08	+	+	Non-1(D1)
1 & 2	PAC2 non-1(D1)	10^−2^ PAC1 non-1(D1)	2(G1)	MB08	+	+	Non-1(D1)
1	Sample 1	wart tissue	2(G1)	MB08	+	+	Non-1(D1)
	Sample 2	wart tissue	1(D1)	MB42	+	+	1(D1)
	Sample 3	wart tissue	18(T1)	MB86	+	+	Non-1(D1)
	Sample 4	wart tissue	38(Nevsehir)	MB56	+	+	Non-1(D1)
	Sample 5	healthy potato	Eersteling	–	–	–	–
	Sample 6	wart tissue	6(O1)	MB10	+	+	Non-1(D1)
	Sample 7	wart tissue	18(T1)	MB86	+	+	Non-1(D1)
	Sample 8	wart tissue	6(O1)	MB10	+	+	Non-1(D1)
	Sample 9	wart tissue	2(G1)	MB08	+	+	Non-1(D1)
	Sample 10	wart tissue	38(Nevsehir)	MB56	+	+	Non-1(D1)
	Sample 11	wart tissue	1(D1)	MB42	+	+	1(D1)
	Sample 12	healthy potato	Eersteling	–	–	–	–
	NIC	healthy potato	Eersteling	–	–	–	–
	PIC	wart tissue	1(D1)	MB42	+	+	1(D1)
	Return Sample	Wart tissue	2(G1)	MB08	+	+	Non-1(D1)
2	Sample 1	5 sps^b^	1(D1)	MB42	Und^c^	Und	Und
	Sample 2	5000 sps	6(O1)	MB10	+	+	Non-1(D1)
	Sample 3	MGW	–	–	–	–	–
	Sample 4	50 sps	6(O1)	MB10	Und	Und	Und
	Sample 5	5 sps	6(O1)	MB10	Und	Und	Und
	Sample 6	500 sps	1(D1)	MB42	+	+	1(D1)
	Sample 7	MGW	–	–	–	–	–
	Sample 8	500 sps	6(O1)	MB10	+	+	Non-1(D1)
	Sample 9	50 sps	1(D1)	MB42	Und	Und	Und
	Sample 10	5000 sps	1(D1)	MB42	+	+	1(D1)
	NIC	MGW	–	–	–	–	–
	PIC	5000 sps	1(D1)	MB42	+	+	1(D1)

a. molecular grade water, b. resting spores per sample (10 μL molecular grade water), c. Undetermined: samples below the limit of detection, and with repeatability scores <100%

In the second TPS round, resting spore suspensions were provided. The unknown sample set consisted of molecular grade water (MGW; Sigma, Saint Louis, USA) used for resting spore suspension preparation, and undiluted (approximately 5.0 × 10^5^ spores mL^−1^) and two 10-fold dilutions of *S. endobioticum* pathotype 1(D1) and 6(O1) resting spore suspensions (Table 1). Resting spores were isolated from fresh warts with 75 μm and 45 μm mesh sieves. A heat treatment (15 min at 95 °C) was performed on the resting spore suspension stocks to render the resting spores non-viable. Preliminary tests performed on heat treated and non-heat treated resting spore suspensions showed that the heat treatment had no effect on PCR success (data not shown). For each sample, 10 μL heat treated resting spore suspensions or MGW was added to a 1.5 mL screw cap tube (VWR, Radnor, USA).

Sample set homogeneity was determined by analysing 10 aliquots per sample using all tests.

### TPS organisation

Participants were provided with most items needed for TPS participation to minimise factors that could influence test performance. In both TPS rounds, participants were provided with positive and negative amplification controls (PAC and NAC), positive and negative isolation controls (PIC and NIC), and 10 unknown samples. In addition, TPS packages contained aliquots of the DNeasy Plant Mini Kit (Qiagen, Hilden, Germany), primers and probes, a return sample, 15 mL MGW for reaction mix preparation, transport documents for the return sample (aliquot of sample wart 2(G1), round 1 only) and an instruction booklet. Test descriptions were provided following the format for EPPO diagnostic protocols to not only determine the test performance, but also the user-friendliness of the test description proposed for the update of EPPO PM 7/28(1). The test description provided to the TPS participants is presented in the supplementary material (SI 1).

Prior to shipment of TPS packages, aliquoted samples, primers, probes and extraction kits were tested for homogeneity. Reagents and extraction kits used for the homogeneity tests were taken from the same batch as provided to the participants. TPS samples were regarded as suitable when resulting in the expected qualitative results and producing Ct values with standard deviations <3.3.

Upon receipt of the TPS package, partners had to send the return sample to the TPS organisers who extracted DNA from the samples and analysed them using the Sendo TaqMan. Ct values obtained from the return samples were used to determine if sample shipment influenced the TPS results.

When TPS partners obtained unclear or contradictory results for the included controls, a spare sample set had to be used to repeat the tests. For each sample analysed, participants had to provide qualitative test results, gel-images for the conventional Sendo PCR and Ct values for the TaqMan tests. In addition participants had to state if the protocols were strictly followed, and which grinding procedure and thermocyclers were used.

### Performance criteria

Using the data generated by TPS participants, diagnostic sensitivity, diagnostic specificity, accuracy, repeatability and robustness were determined for each test-matrix combination. Data from partners that failed to produce correct results for the provided controls were excluded from the analysis. Positive agreement (PA), negative agreement (NA), positive deviation (PD) and negative deviation (ND) (EPPO 2014a, b) of results provided by TPS participants relative to the assigned values based on the homogeneity tests were calculated. Diagnostic sensitivity (PA/(PA + ND), diagnostic specificity (NA/(NA + PD) and accuracy ((PA + NA)/(PA + NA + PD + ND)) are expressed in percentages and provide insight in false negative results, false positive results and the overall performance of a test respectively. Each TPS participant received multiple aliquots of the same original sample. Qualitative results of these biological duplicates and triplicates were used to calculate the repeatability per TPS partner per test-matrix combination. Robustness was determined by inventorying variations to the protocols (i.e. disruption methods and thermocyclers used) to determine if they influenced test accuracy. For disruption methods, qualitative and quantitative results of the Sendo TaqMan were used, whereas for thermocyclers qualitative results of the positive amplification controls were used to determine the robustness.

For wart material the analytical sensitivity was determined with dilutions of DNA from infected warts in DNA from healthy potato using seven samples covering five pathotypes. For resting spore suspensions the analytical sensitivity was determined with dilutions of resting spore suspensions using five samples covering two pathotypes. As the presence of the non-culturable pathogen cannot be quantified from wart material, a relative infection rate was used to express the limit of detection (LOD). Naturally infected potato wart pieces were regarded to have a relative infection rate of 100%. For resting spore suspensions, the amount of resting spores per sample is used to express the LOD. Wart material of fifteen *S. endobioticum* strains covering five different pathotypes were analysed to determine the analytical specificity (i.e. performance of a test with regard to cross-reactions with non-targets; EPPO 2017) of the different tests. Materials used to determine the analytical sensitivity and analytical specificity were tested as described in the supplementary information (SI 1).

## Results

### Homogeneity and stability results

Potato wart sample sets used in TPS round 1 produced homogenous test results for all samples in all tests (SI table 1). Mean Ct values ranged from 15.5 to 19.6 with standard deviations ranging from 1.0 to 2.2. Resting spore suspensions (round 2) produced homogenous test results with the Sendo TaqMan for the undiluted and 10× diluted samples for both pathotypes, and with the 1(D1) TaqMan for undiluted samples of pathotype 6(O1). Other sample-test combinations failed to produce the expected qualitative results for all aliquots tested. In the analysis of the TPS results, samples with 5000 and 500 resting spores per sample were regarded positive for all tests and were used to determine the performance criteria of the tests.

Return samples analysed with the Sendo TaqMan were used to determine the sample stability. Analysis of the return samples produced Ct values similar to the Ct values obtained for the homogeneity tests indicating that sample shipment did not negatively influence the TPS results (Ct_mean homogeneity tests_: 20.7, Ct_mean return samples_: 19.6, *p* Students T-test: 0.284).

### Sendo TaqMan: Ct cut-off value

**Fig. 1:**
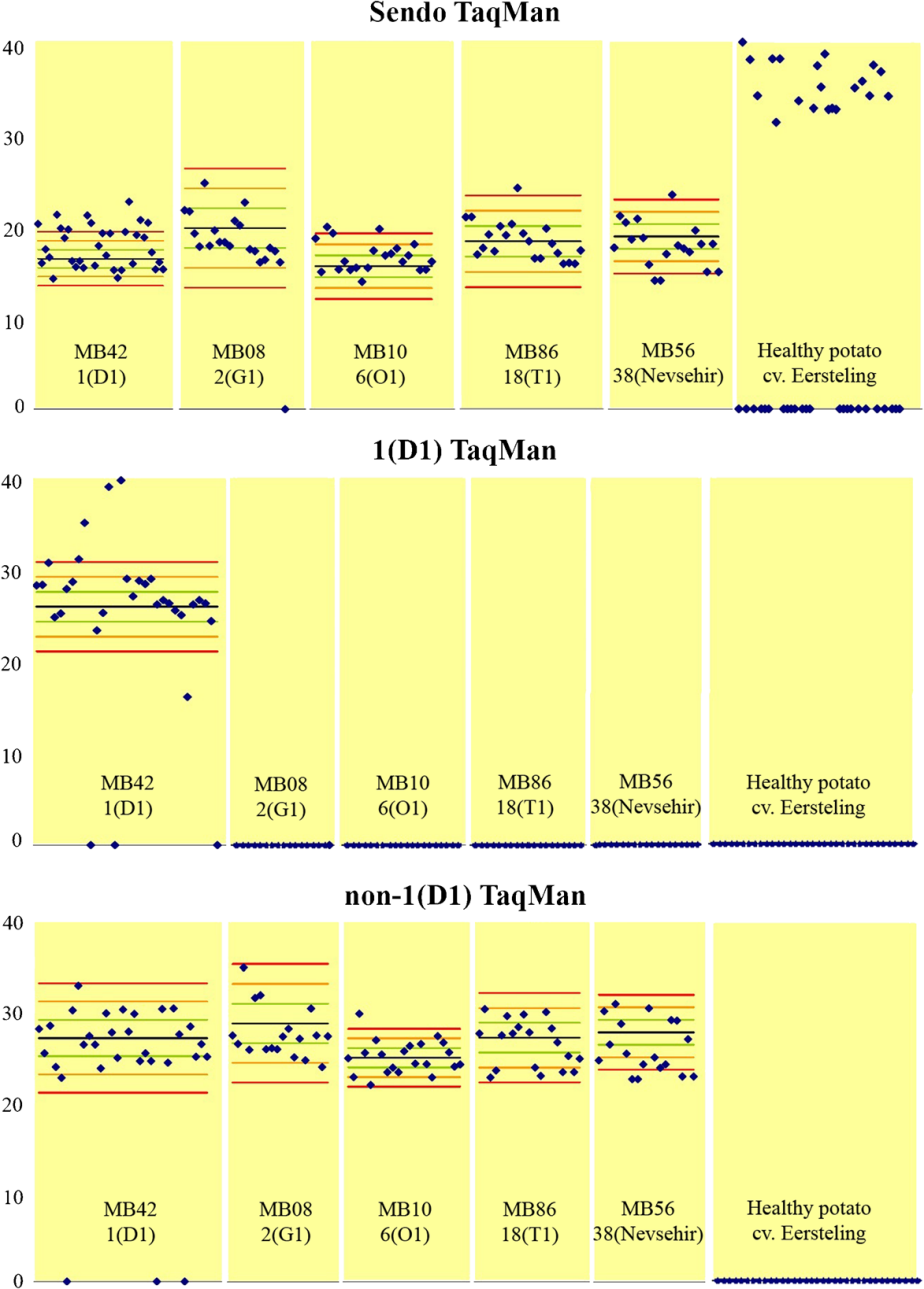
TPS round 1 (wart material) Ct values obtained by TPS participants (♦) for the Sendo TaqMan, and the 1(D1) TaqMan (pathotype 1(D1) test, and non-pathotype 1(D1) test). Horizontal blue lines represent the mean Ct values obtained from the homogeneity tests. Corresponding ±1 SD, ±2 SD and ±3 SD values are represented by green, orange and red horizontal lines, respectively. Negative samples are assigned the value “0”

Analysis of TPS round 1 data showed that late Ct values were obtained in the Sendo TaqMan in 20 of the 44 healthy potato samples (Fig 1). A Ct cut-off value was determined to distinguish false positive results from truly positive samples for this test-matrix combination. Qualitative data provided by TPS participants and homogeneity results were used to calculate the mean false-positive Ct value and corresponding standard deviation. Three standard deviations were subtracted from the mean false positive Ct value resulting, after rounding down to the nearest natural number, in a Ct cut-off value of 30. Performance criteria presented in this paper are based on the cut-off value of 30 unless stated otherwise. A Ct cut-off value is not needed when testing resting spores suspensions.

### Diagnostic sensitivity, diagnostic specificity and accuracy

Using wart material for *S. endobioticum* detection, diagnostic sensitivity (the percentage of sample with presence of the target that test positive) values ranging from 95.4% to 97.2% are obtained (Table 2). For diagnostic specificity (the percentage of samples with absence of the target that test negative) the Sendo PCR, Sendo TaqMan and 1(D1) TaqMan yield 100% correct results. No significant differences (2-sample binomial tests) for diagnostic sensitivity (*p* ≥ 0.344), diagnostic specificity (*p* = 1.0) and accuracy (*p* ≥ 0.461) are found between the different tests when testing wart material. All wart samples analysed resulted in correct results for the internal COX control with an average Ct value of 19.3 and standard deviation of 1.9.

**Table 2:** Diagnostic sensitivity, diagnostic specificity and overall accuracy values obtained using TPS results

TPS round	Test	DSens^a^	DSpec^b^	Acc^c^
1 (wart material)	Sendo PCR	96.3%	100%	97.2%
	Sendo TaqMan	97.2%	100%	97.9%
	1(D1) TaqMan	95.4%	100%	96.5%
2 (resting spores)	Sendo PCR	68.0%	100%	73.9%
	Sendo TaqMan	76.7%	100%	85.4%
	1(D1) TaqMan	45.7%	77.1%	61.4%

a. diagnostic sensitivity, b. diagnostic specificity, c. accuracy

When testing resting spores, the highest values for diagnostic sensitivity, diagnostic specificity and overall accuracy are obtained with the Sendo TaqMan (Table 2). The Sendo PCR and Sendo TaqMan significantly outperform the 1(D1) TaqMan for diagnostic sensitivity (2-sample binomial test, *p* ≤ 0.05) and diagnostic specificity (*p* = 0.003). The Sendo TaqMan significantly outperforms both the Sendo PCR and 1(D1) TaqMan for overall accuracy (*p* < 0.030).

### Repeatability

Biological duplicates and triplicates tested by TPS partners were used to calculate the repeatability of the different tests per participant for each test matrix (SI Table 2). For TPS round 1, results of 13 partners were included in the analysis, and a total of 52 repeatability samples were analysed. Similar repeatability results are obtained for all three tests. With an average repeatability of 98%, the TPS participants produced repeatable results when analysing wart material. For the second TPS round, 14 to 28 repeatability samples tested for the Sendo PCR (14 partners), Sendo TaqMan (12 partners), and 1(D1) TaqMan (seven partners) were analysed. When testing resting spore suspensions with 5000 spores per sample, the Sendo TaqMan and Sendo PCR significantly outperform the 1(D1) TaqMan (2-sample binomial test, *p* ≤ 0.029) with an average 83% repeatability. Even though the Sendo PCR statistically outperforms the 1(D1) TaqMan, the average reproducibility of both test is poor when analysing resting spore suspensions (64% and 29% respectively).

### Robustness

In TPS round 1, eleven participants specified the disruption method used (i.e. manual versus mechanical disruption). Two partners manually grinded samples preceding the DNA extraction, whereas nine partners indicated they used a mechanical disruption method. Samples tested as biological duplicate or triplicate (i.e. wart 1(D1), and healthy potato) were used to determine the qualitative and quantitative influence of the disruption method applied. No significant differences are found based on qualitative test results. Manual disruption of the samples resulted in higher Ct values for the Sendo TaqMan (+3.1) and 1(D1) TaqMan (+3.4). Data obtained in the second TPS round could not be used to determine the robustness when using the tests with resting spores as starting material as only mechanical disruption methods were used (SI Table 3).

Expected qualitative test results for the conventional PCR test were obtained using the following thermocyclers: Peltier PTC-200 (MJ research), GeneAmp PCR System 9700 (Applied Biosystems), GeneAmp PCR System 2720 (Applied Biosystems), Mastercycler personal (Eppendorf), C1000 (Bio-Rad), Veriti 96-well thermal cycler (Applied Biosystems). Expected qualitative test results for the real-time PCR tests were obtained using the following real-time PCR systems: 7300 Real-Time PCR System (Applied Biosystems), 7900HT Fast real-time PCR system (Applied Biosystems), ABI 7500 Real-time PCR system (Applied Biosystems), CFX96 (Bio-Rad), Mastercycler ep realplex (Eppendorf), and Stratagene Mx3005P (Agilent genomics).

### Analytical sensitivity

The lowest amount of the target at which all samples produce a positive results is regarded as the LOD which were determined for both test matrices at intra-laboratory level (SI Table 4). When using wart material as input, the Sendo PCR, Sendo TaqMan, Bonants test for pathotype 1(D1) samples, and Bonants test for non-pathotype 1(D1) samples have a LOD at a relative infection rate of 1% (i.e. a 100× dilution of a naturally infected wart). Using resting spore suspensions, only the Sendo TaqMan, and the non-1(D1) TaqMan produced consistent results for all subsamples at 500 and 5000 resting spores per 10 μL sample respectively. The Sendo PCR, and the 1(D1) TaqMan were not sensitive enough to detect the pathogen in all subsamples with 5000 resting spores per 10 μL sample. The LOD for the latter two tests lies higher than 5000 spores per 10 μL sample.

### Analytical specificity

Analytical specificity is the ability of a test to detect a particular target, rather than others. In case of the Sendo PCR and Sendo TaqMan, *S. endobioticum* (covering all pathotypes) is the target, whereas in the 1(D1) TaqMan only *S. endobioticum* pathotype 1(D1) strains are the target. In the first two tests, healthy potato material serves as non-target material, whereas in the third test both healthy potato material and *S. endobioticum* strains of pathotypes other than 1(D1) are non-targets. Wart material of 15 strains was analysed to determine their reaction in the different tests (SI Table 5). For the Sendo PCR and Sendo TaqMan all strains produced results as expected; i.e. positive in case *S. endobioticum* was present in the sample. For the 1(D1) TaqMan however, one Swedish pathotype 1(D1) isolate produced a result consistent with non-pathotype 1(D1) samples; i.e. a false negative result. Other isolates produced results as expected in the 1(D1) TaqMan.

## Discussion and Conclusions

Three tests were selected for the detection and identification of *S. endobioticum*, the causal agent of potato wart disease. Tests were validated in an international TPS with fifteen participants for two test matrices: warted potato tissue (round 1), and resting spore suspensions (round 2). Guidance in the EPPO standard on organisation of interlaboratory comparison studies (EPPO, 2014b) was found very helpful in setting up the TPS. Partners had to analyse 10 samples per TPS round. When unclear or inconsistent results were obtained, the analysis had to be repeated with a back-up sample set. Datasets with incorrect results for the control samples were excluded from the analysis resulting in 13 datasets in round 1 and 14 (Sendo PCR), 13 (Sendo TaqMan), and 7 (1(D1) TaqMan) datasets in round 2.

Several TPS partners generated late Ct values for some of the healthy potato samples tested. A Ct cut-off value was determined for the Sendo TaqMan to eliminate late Ct values without introducing false negative results. These late Ct values could be the result of contamination or non-specific annealing of primers and probe. During the preparation of the TPS, late Ct values in healthy potato tissue were observed only once. After finalisation of the Euphresco Sendo project, researchers at ILVO compared the Sendo TaqMan with a real-time PCR test targeting the small ribosomal subunit (18S) described by Smith *et al*. (2014), which was not available at the time of the TPS setup, on extracts obtained with zonal centrifugation (personal communication Kurt Heungens, ILVO, Belgium). The Sendo TaqMan produced late Ct values for some truly negative samples, which could not be reproduced with the Smith test. With the Smith test producing slightly lower Ct values in general, this suggests late Ct values are the result of non-specific annealing of primers and probes in the Sendo TaqMan rather than as a result of contamination. We propose to include the cut-off value in the EPPO standard. No false negative results were obtained when using the cut-off value. Laboratories implementing the Sendo TaqMan have to determine the need of a Ct cut-off value for their diagnostic workflow through the process of verification.

When using the tests for detection and identification of *S. endobioticum* in warted potato tissue (TPS round 1), no significant differences were observed for diagnostic sensitivity, diagnostic specificity and overall accuracy and the tests are regarded equal. Also, the tests show an equal performance in terms of analytical sensitivity using this test matrix. All tests were found to be robust for the disruption method used.

The second TPS round proved to be more challenging than the first since the sample set provided contained resting spore suspensions close or below the limit of detection. When using the tests for detection and identification of *S. endobioticum* in resting spore suspensions, the Sendo PCR and Sendo TaqMan significantly outperformed the 1(D1) TaqMan for diagnostic sensitivity and diagnostic specificity. For overall accuracy, the Sendo TaqMan significantly outperforms both the Sendo PCR and 1(D1) TaqMan. Using the 1(D1) TaqMan for pathotype identification at low levels of the target proved to be difficult. Under the conditions used in the TPS we would recommend to use caution when testing resting spore suspensions below 5000 spores per sample.

For the determination of analytical specificity, samples used were limited to different *S. endobioticum* pathotypes and healthy potato as no other *Synchytrium* spp. were available to us. It is not likely that the symptoms caused by potato wart disease are also induced by closely related *Synchytrium* species as they are highly specialised for certain hosts. Wart disease symptoms could be confused with pseudo-wart: a proliferation of eyes that may be a physiological response, a varietal response, or could be induced by chemical factors. In essence, potatoes with pseudo-wart are healthy potatoes. False non-pathotype 1(D1) results were obtained with Swedish pathotype 1(D1) isolate (MB69) using the 1(D1) TaqMan. Bonants *et al*. (2015) obtained similar results with some pathotype 1(D1) isolates originating from outside the Netherlands and Germany. The pathotype 1(D1) associated SNP that lies at the basis of the 1(D1) TaqMan design was identified using Dutch and German 1(D1) isolates. This means that for diagnostic purposes, only pathotype 1(D1) positive results produced by the 1(D1) TaqMan can be used for molecular 1(D1) identification. Strains identified as pathotype 1(D1) using a bioassay can produce non-pathotype 1(D1) results in the 1(D1) TaqMan.

In addition, the 1(D1) TaqMan test was found difficult to interpret as, apart from Ct values, participants had to identify pathotype 1(D1) and non-pathotype 1(D1) specific amplification plots as described by Bonants *et al.* (2015). This proved particularly challenging for resting spore suspensions with low amount of target. Some TPS partners indicated that, under their conditions, Ct values were as expected but that the obtained amplification plots were different compared to the expected reactions. The TPS organisers and some TPS partners also found slight differences in the amplification plots for some *S. endobioticum* collection items compared to the results published by Bonants *et al.*. Real-time PCR machines, and in particular the ramp rates could have an influence on the amplification efficiency and the corresponding shape of the real-time PCR amplification curves. This aspect has not been further investigated under the TPS. The added value of the non-1(D1) reaction was questioned as it was found to be confusing. Improved user-friendliness of the 1(D1) TaqMan can be achieved by using the 1(D1) reaction without the non-1(D1) reaction in conjunction with one of the generic *S. endobioticum* tests. It is this combination we propose to include in the update of EPPO PM7/28.

As goes for all potato wart disease studies, having access to sufficient and well characterised isolates covering a broad geographical range is challenging because of the low outbreak frequency for this pest, the fact that it is difficult to maintain in collections, and the different pathotyping methods used in different laboratories. However, having sufficient and well characterised isolates covering a broad geographical range is paramount for reliable development and validation of diagnostic tests. The *Synchytrium endobioticum* community would strongly benefit from a centralised repository for collection material that maintains the material and keeps track of its “genealogy”. Euphresco partners could play a role in addressing this when new potato wart disease initiatives are launched within this research framework. For future diagnostic wart disease projects, recently published tests for molecular *S. endobioticum* detection (Smith *et al*., 2014), and genotype identification (Gagnon *et al*. 2016) should be considered.

## Electronic supplementary material

ESM 1 (DOCX 64 kb)
