# Supplementary material for: Euphresco Sendo: An international laboratory comparison study of molecular tests for Synchytrium endobioticum detection and identification
Source: Eur J Plant Pathol. 2018 Feb 7;151(3):757–66. doi: 10.1007/s10658-017-1411-6 (PMC6445494; doi:10.1007/s10658-017-1411-6)
Supplement: Supplementary file 1 — (DOCX 64 kb) [file 10658_2017_1411_MOESM1_ESM.docx]

**Supplementary information**

Euphresco Sendo: An international laboratory comparison study of molecular tests for *Synchytrium endobioticum* detection and identification

van de Vossenberg *et al*.

**1. Protocols in EPPO diagnostic standard format as presented to TPS participants.**

Below, test descriptions as provided to the TPS partners are shown. As the 1(D1) and non-1(D1) Taqman tests were not published at the time of the TPS, primer and probe sequences were not provided to the TPS partners. As a result of the TPS, changes were made to the protocols before submitting them to the EPPO secretariat to consider them for the update of PM7/28(1) *Synchytrium endobioticum*. For the final version accepted by EPPO please consult PM7/28(2) .

As is customary for EPPO protocols, tests are described in great detail including reagents and equipment used. The description of reagents and equipment does not exclude the use of others. Labs implementing these tests using other reagents or equipment have to verify the altered test conditions do not negatively influence the test results.

**Detection of *S. endobioticum* in potato wart material using conventional PCR**

1. **General Information**
   1. Detection of *S. endobioticum* in potato warts using conventional PCR developed by Levesque *et al.* 2001
   2. The conventional PCR was first published by Levesque *et al.* in 2001, but described in detail by van den Boogert *et al.* in 2005. The PCR reaction mix was updated by NPPO-NL in 2013 and validated in an international test performance study as such. Any reference to this study???
   3. Primers F49 (5’-CAACACCATGTGAACTG-3’) and R502 (5’‑ACATACACAATTCGAGTTT-3’) amplify 472 bp of the internal transcribed spacer (ITS) region.
   4. Amplification is performed in a thermal cycler with heated lid, e.g. T100 Thermal cycler (Bio-Rad). Why this apparatus? Not the one in the original paper? Would be better to give the requirements of the equipement (e.g. ramp, block system, no carrousel…)
2. **Methods**
   1. Nucleic Acid Extraction and Purification

Potato wart material (max. 100 mg) is extracted using a modified Plant Tissue mini protocol from the DNeasy® Plant Mini Kit (Qiagen).

- - 1. Transfer wart material (max. 100 mg) to a 2 mL microcentrifuge tube. Add two steel beads (e.g. 4 mm diameter) to the vial when mechanical disruption is used
    2. Add 400 μL Buffer AP1 and 4 μL RNase A stock solution (100 mg/mL). Buffer AP1 may form precipitates upon storage. If necessary, warm to 65°C to re-dissolve
    3. Disrupt the sample either manually (e.g. with a micro pestle) or mechanically (e.g. with a BeadBeater or TissueLyser)
    4. Incubate the mixture for 10 min at 65°C. Mix 2 or 3 times during incubation by inverting tube
    5. Centrifuge 1 min at 20,000 x g and transfer the supernatant (= lysate) to a new 1.5 mL microcentrifuge tube
    6. Add 130 μL Buffer P3 to the lysate and mix.
    7. Incubate for 5 min on ice
    8. Centrifuge the lysate for 5 min at 20,000 x g
    9. Pipet the lysate into the QIAshredder Mini spin column (lilac) placed in a 2 mL collection tube
    10. Centrifuge for 2 min at 20,000 x g
    11. Transfer 450 μL of the flow-through fraction from step 10 into a new 1.5 mL microcentrifuge tube
    12. Add 675 μL (1.5 volumes) of Buffer AW1 to the cleared lysate, and mix by pipetting.
    13. Pipet 650 μL of the mixture from step 12, including any precipitate that may have formed, into the DNeasy Mini spin column placed in a 2 mL collection tube.
    14. Centrifuge for 1 min at 6000 x g
    15. Place the DNeasy Mini spin column into a new 2 mL collection tube and repeat step 13 with remaining sample.
    16. Place the DNeasy Mini spin column into a new 2 mL collection tube, add 500 μL Buffer AW2,
    17. Centrifuge for 1 min at 6000 x g.
    18. Place the DNeasy Mini spin column into a new 2 mL collection tube, add 500 μL Buffer AW2 to the DNeasy Mini spin column.
    19. Centrifuge for 2 min at 20,000 x g to dry the membrane.
    20. Transfer the DNeasy Mini spin column to a 1.5 mL microcentrifuge tube, and pipet 50 μL Buffer AE directly onto the DNeasy membrane. Incubate for 5 min at room temperature (15 - 25°C).
    21. Centrifuge for 1 min at 6000 x g. The flow through contains the extracted DNA.
    22. After DNA extraction, no additional DNA clean-up is required. Either use extracted DNA immediately or store it at –20 °C until use.
  1. Conventional PCR
     1. Master Mix conventional PCR

| Reagent | Working concentration | Volume per reaction (µL) | Final concentration |
| --- | --- | --- | --- |
| Molecular grade water^*^ | N.A. | 13.8 | N.A. |
| Colorless GoTaq Flexi buffer (Promega) | 5x | 5.0 | 1x |
| MgCl_2_ (Promega) | 25 mM | 1.5 | 1.5 mM |
| dNTP's (Promega) | 10 mM each | 0.5 | 0.2 mM |
| Primer F49 | 10 µM | 1.5 | 600 nM |
| Primer R502 | 10 µM | 1.5 | 600 nM |
| GoTaq DNA Polymerase (Promega) | 5 U/µL | 0.2 | 1 Unit |
| Subtotal |  | 24.0 |  |
| Genomic DNA extract |  | 1.0 |  |
| Total |  | 25.0 |  |

*Molecular grade water should be used preferably. Alternatively sterile (autoclaved or 0.45 µm filtered), purified (deionised or distilled) and nuclease-free water can be used.

- - 1. PCR conditions: 2 min 95 °C, 35x (30 sec 95 °C, 30 sec 57 °C, 30 sec 72 °C), 5 min 72 °C, quick cooling to room temperature

1. **Essential Procedural Information**
   1. Controls

For a reliable test result to be obtained, the following (external) controls should be included for each series of nucleic acid extraction and amplification of the target organism and target nucleic acid, respectively

- Negative isolation control (NIC) to monitor contamination during nucleic acid extraction: DNA extraction from healthy potato material (max. 100 mg)
- Positive isolation control (PIC) to ensure that nucleic acid of sufficient quantity and quality is isolated: DNA extraction from *S. endobioticum* (e.g. pathotype 1) infected potato wart material (max. 100 mg).
- Negative amplification control (NAC) to rule out false positives due to contamination during the preparation of the reaction mix: amplification of molecular grade water that was used to prepare the reaction mix.
- Positive amplification control (PAC) to monitor the efficiency of the amplification: amplification of undiluted DNA extracted from *S. endobioticum* (e.g. pathotype 1) infected potato wart material (max. 100 mg).
  1. Interpretation of results

In order to assign results from this test the following criteria should be followed:

*Verification of the controls*

- NIC and NAC should produce no amplicons
- PIC and PAC should produce amplicons of 472 bp

*When these conditions are met:*

- A test will be considered positive if amplicons of 472 bp are produced
- A test will be considered negative, if it produces no band or a band of a different size
- Tests should be repeated if any contradictory or unclear results are obtained

**Detection of *S. endobioticum* in potato wart material and winter spore suspension using real-time PCR**

1. **General Information**
   1. Detection of *S. endobioticum* in potato warts and winter spore suspension using real-time PCR developed by van Gent-Pelzer *et al*. (2010)
   2. The test is designed to amplify 84 bp of the internal transcribed spacer 2 (ITS2) sequence of *S. endobioticum* and 79 bp of the cytochrome oxidase subunit 1 (COX) of plant DNA as internal control.
   3. Forward primer SendoITS2F (5’-TTTTTACGCTCACTTTTTTTAGAATGTT-3’); reverse primer SendoITS2R (5’-CTGCCTCACACACCACATACA-3’) Sendo probe2 (5’-AATTCGAGTTTGTCAAAAGGTGTTTGTTGTGG-3’ ) FAM label and Eclipse Dark Quencher (EDQ); forward primer COX F (5’-CGTCGCATTCCAGATTATCCA-3’); reverse primer COX RW (5’- CAACTACGGATATATAAGRRCCRRAACTG-3’); probe COXSOL 1511T (5’-AGGGCATTCCATCCAGCGTAAGCA–3) Yakima Yellow label and Black Hole Quencher 1 (BHQ1).
   4. Amplification is performed in a real-time PCR thermal cycler with heated lid, e.g. CFX96 (Bio-Rad).
2. **Methods**
   1. Nucleic Acid Extraction and Purification
      1. Potato wart material (max. 100 mg) or winter spore suspension (10 μL) is extracted using a modified Plant Tissue mini protocol from the DNeasy® Plant Mini Kit (Qiagen). See also section 2.1 of appendix 1.
      2. After DNA extraction, no additional DNA clean-up is required. Either use extracted DNA immediately or store it at –20 °C until use.
   2. Real-time PCR van Gent-Pelzer *et al*. (2010)
      1. Two simplex reactions are prepared; one for *S. endobioticum* detection, and one for amplification of the Plant COX gene as internal control.
      2. Master Mix real-time PCR *S. endobioticum* detection

| Reagent | Working concentration | Volume per reaction (µL) | Final concentration |
| --- | --- | --- | --- |
| Molecular grade water^*^ | N.A. | 10.25^&^ | N.A. |
| 2x Premix Ex Taq (TaKaRa) | 2x | 15.0 | 1x |
| ROX Reference Dye/Dye II (TaKaRa) | Use when needed ^#^ | | |
| SendoITS2F | (10 µM) | 0.75 | 250 nM |
| SendoITS2R | (10 µM) | 0.75 | 250 nM |
| Sendo probe2 | (10 µM) | 0.25 | 83 nM |
| Subtotal |  | 27.0 |  |
| Genomic DNA extract |  | 3.0 |  |
| Total |  | 30.0 |  |

* Molecular grade water should be used. Alternatively sterile (autoclaved or 0.45 µm filtered), purified (deionised or distilled) and nuclease-free water can be used.

& The Molecular Grade Water volume is reduced to 9.65 µL when ROX Reference Dye/Dye II is used.

# The ROX Reference Dye/Dye II is used for normalization of the fluorescent signal when working with Applied Biosystems (AB) real-time PCR instruments. For AB 7000/7700/7900HT and 7300 Real-Time PCR Systems, use 0.6 µL ROX Reference Dye (50x), final concentration 1x. For the AB 7500 Real-Time PCR System use 0.6 µL ROX Reference Dye II (50x), final concentration 1x. When the ROX Reference Dye or Dye II is used, reduce the volume of molecular grade water with 0.6 µL per reaction.

- - 1. Master Mix real-time PCR for plant DNA amplification

| Reagent | Working concentration | Volume per reaction (µL) | Final concentration |
| --- | --- | --- | --- |
| Molecular grade water^*^ | N.A. | 10.5^&^ | N.A. |
| 2x Premix Ex Taq (TaKaRa) | 2x | 15.0 | 1x |
| ROX Reference Dye/Dye II (TaKaRa) | Use when needed ^#^ | | |
| COX F | (10 µM) | 0.6 | 200 nM |
| COX RW | (10 µM) | 0.6 | 200 nM |
| COXSOL 1511T | (10 µM) | 0.3 | 100 nM |
| Subtotal |  | 27.0 |  |
| Genomic DNA extract |  | 3.0 |  |
| Total |  | 30.0 |  |

* Molecular grade water should be used. Alternatively sterile (autoclaved or 0.45 µm filtered), purified (deionised or distilled) and nuclease-free water can be used.

& The Molecular Grade Water volume is reduced to 9.9 µL when ROX Reference Dye/Dye II is used.

# The ROX Reference Dye/Dye II is used for normalization of the fluorescent signal when working with Applied Biosystems (AB) real-time PCR instruments. For AB 7000/7700/7900HT and 7300 Real-Time PCR Systems, use 0.6 µL ROX Reference Dye (50x), final concentration 1x. For the AB 7500 Real-Time PCR System use 0.6 µL ROX Reference Dye II (50x), final concentration 1x. When the ROX Reference Dye or Dye II is used, reduce the volume of molecular grade water with 0.6 µL per reaction.

- - 1. PCR conditions: 95 ºC for 10 min, 40 cycles of 95 ºC for 15 sec and 60 ºC for 1 min

1. **Essential Procedural Information**
   1. Controls

For a reliable test result to be obtained, the following (external) controls should be included. These are used for each series of nucleic acid extraction and amplification of the target organism:

- Negative isolation control (NIC) to monitor contamination during nucleic acid extraction: DNA extraction from healthy potato material (max. 100 mg)
- Positive isolation control (PIC) to ensure that nucleic acid of sufficient quantity and quality is isolated: DNA extraction from *S. endobioticum* (e.g. pathotype 1) infected potato wart material (max. 100 mg).
- Negative amplification control (NAC) to rule out false positives due to contamination during the preparation of the reaction mix: amplification of molecular grade water that was used to prepare the reaction mix.
- Two positive amplification controls (PAC1 and PAC2) to monitor the efficiency of the amplification: amplification of undiluted and 100x diluted DNA extracted from *S. endobioticum* (e.g. pathotype 1) infected potato wart material (max. 100 mg).

In addition to the external positive controls (PIC, PAC1 and PAC2), an internal positive isolation control is used to monitor each individual sample separately (specific amplification of plant COX gene). The use of this internal control is not necessary in case of *S. endobioticum* detection in winter spore suspension.

- 1. Interpretation of results

The cycle cut off value for both *S. endobioticum* and the Plant COX gene is set at 40, and was obtained using the equipment, materials and chemistry used as described in this appendix. When necessary the Ct cut off value should be determined for the required control.

The cycle cut off value needs to be verified in each laboratory when implementing the test for the first time. To assign results from real-time PCR-based tests the following criteria should be followed:

*Verification of the controls*

- NAC should be negative (Ct > cut off) for both *S. endobioticum* and the Plant COX gene.
- NIC should be negative (Ct > cut off) for *S. endobioticum*, and for the Plant COX gene produce an exponential amplification curve with a Ct value below the cut off value.
- PIC, PAC1 and PAC2 should produce an exponential amplification curve, and a Ct value below the cut off value for both *S. endobioticum* and the Plant COX gene.

*When these conditions are met:*

- A test will be considered positive if it produces an exponential amplification curve, and a Ct value below the cut off value for *S. endobioticum*.
- A test will be considered negative, if it produces no exponential amplification curve and/or a Ct value equal or above the cut off value for *S. endobioticum,* and for the Plant COX gene an exponential amplification curve, and a Ct value below the cut off value.
- Tests should be repeated if any contradictory or unclear results are obtained.

**Identification of *S. endobioticum* pathotype 1 in potato wart material using real‑time PCR**

1. **General Information**
   1. Detection of *S. endobioticum* in potato warts and winter spore suspension using real-time PCR developed by Bonants *et al*. (unpublished) and optimised by HLB, Wijster, the Netherlands.
   2. The test is designed to amplify 122 bp of a region containing a pathotype 1(D1) versus non- 1(D1) pathotype specific single nucleotide polymorphism (SNP) identified by CroPs analysis. Both *S. endobioticum* 1(D1) and non-1(D1) strains can be identified in a duplex reaction. In addition, 79 bp of the cytochrome oxidase subunit 1 (COX) of plant DNA can be amplified as internal control.
   3. Forward primer Fw_L1 + 2; reverse primer Rv_L1+1 probe P1_L1 VIC VIC label and Black Hole Quencher 1 (BHQ1) quencher; probe P2-18_L1 A546 FAM label en BHQ1 quencher; forward primer COX F (5’‑CGTCGCATTCCAGATTA TCCA-3’); reverse primer COX RW (5’‑CAACTACGGATATATAAGRRCCRRAA CTG-3’); probe COXSOL 1511T (5’-AGGGCATTCCATCCAGCGTAAGCA–3) Yakima Yellow label and BHQ1 (BHQ1)
   4. Amplification is performed in a real-time PCR thermal cycler with heated lid, e.g. CFX96 (Bio-Rad).
2. **Methods**
   1. Nucleic Acid Extraction and Purification
      1. Potato wart material (max. 100 mg) is extracted using a modified Plant Tissue mini protocol from the DNeasy® Plant Mini Kit (Qiagen). See also section 2.1 of appendix 1.
      2. After DNA extraction, no additional DNA clean-up is required. Either use extracted DNA immediately or store it at –20 °C until use.
   2. Real-time PCR Bonants *et al*. (unpublished)
      1. One duplex and one simplex reactions is prepared; one for *S. endobioticum* 1(D1) and non-1(D1) identification, and one for amplification of the Plant COX gene as internal control.
      2. Master Mix real-time PCR *S. endobioticum* 1(D1) and non-1(D1) identification

| Reagent | Working concentration | Volume per reaction (µL) | Final concentration |
| --- | --- | --- | --- |
| Molecular grade water^*^ | N.A. | 9.75^&^ | N.A. |
| 2x Premix Ex Taq (TaKaRa) | 2x | 15.0 | 1x |
| ROX Reference Dye/Dye II (TaKaRa) | Use when needed ^#^ | | |
| Fw_L1 + 2 | (10 µM) | 0.75 | 250 nM |
| Rv_L1+1 | (10 µM) | 0.75 | 250 nM |
| P1_L1 VIC | (10 µM) | 0.25 | 83 nM |
| P2-18_L1 A546 | (10 µM) | 0.5 | 166 nM |
| Subtotal |  | 27.0 |  |
| Genomic DNA extract |  | 3.0 |  |
| Total |  | 30.0 |  |

* Molecular grade water should be used. Alternatively sterile (autoclaved or 0.45 µm filtered), purified (deionised or distilled) and nuclease-free water can be used.

& The Molecular Grade Water volume is reduced to 9.15 µL when ROX Reference Dye/Dye II is used.

# The ROX Reference Dye/Dye II is used for normalization of the fluorescent signal when working with Applied Biosystems (AB) real-time PCR instruments. For AB 7000/7700/7900HT and 7300 Real-Time PCR Systems, use 0.6 µL ROX Reference Dye (50x), final concentration 1x. For the AB 7500 Real-Time PCR System use 0.6 µL ROX Reference Dye II (50x), final concentration 1x. When the ROX Reference Dye or Dye II is used, reduce the volume of molecular grade water with 0.6 µL per reaction.

- - 1. Master Mix real-time PCR for plant DNA amplification

| Reagent | Working concentration | Volume per reaction (µL) | Final concentration |
| --- | --- | --- | --- |
| Molecular grade water^*^ | N.A. | 10.5^&^ | N.A. |
| 2x Premix Ex Taq (TaKaRa) | 2x | 15.0 | 1x |
| ROX Reference Dye/Dye II (TaKaRa) | Use when needed ^#^ | | |
| COX F | (10 µM) | 0.6 | 200 nM |
| COX RW | (10 µM) | 0.6 | 200 nM |
| COXSOL 1511T | (10 µM) | 0.3 | 100 nM |
| Subtotal |  | 27.0 |  |
| Genomic DNA extract |  | 3.0 |  |
| Total |  | 30.0 |  |

* Molecular grade water should be used. Alternatively sterile (autoclaved or 0.45 µm filtered), purified (deionised or distilled) and nuclease-free water can be used.

& The Molecular Grade Water volume is reduced to 9.9 µL when ROX Reference Dye/Dye II is used.

# The ROX Reference Dye/Dye II is used for normalization of the fluorescent signal when working with Applied Biosystems (AB) real-time PCR instruments. For AB 7000/7700/7900HT and 7300 Real-Time PCR Systems, use 0.6 µL ROX Reference Dye (50x), final concentration 1x. For the AB 7500 Real-Time PCR System use 0.6 µL ROX Reference Dye II (50x), final concentration 1x. When the ROX Reference Dye or Dye II is used, reduce the volume of molecular grade water with 0.6 µL per reaction.

- - 1. PCR conditions: 95 ºC for 10 min, 40 cycles of 95 ºC for 15 sec and 60 ºC for 1 min

1. **Essential Procedural Information**
   1. Controls

For a reliable test result to be obtained, the following (external) controls should be included. These are used for each series of nucleic acid extraction and amplification of the target organism:

- Negative isolation control (NIC) to monitor contamination during nucleic acid extraction: DNA extraction from healthy potato material (max. 100 mg)
- Positive isolation control (PIC) to ensure that nucleic acid of sufficient quantity and quality is isolated: DNA extraction from *S. endobioticum* (e.g. pathotype 1) infected potato wart material (max. 100 mg).
- Negative amplification control (NAC) to rule out false positives due to contamination during the preparation of the reaction mix: amplification of molecular grade water that was used to prepare the reaction mix.
- Two positive amplification controls (PAC1 and PAC2) to monitor the efficiency of the amplification of *S. endobioticum* pathotype 1(D1): amplification of undiluted and 100x diluted DNA extracted from *S. endobioticum* 1(D1) infected potato wart material (max. 100 mg).
- Two positive amplification controls (PAC1 and PAC2) to monitor the efficiency of the amplification of non-pathotype 1(D1`) *S. endobioticum* strains amplification of undiluted and 100x diluted DNA extracted from non-1(D1) *S. endobioticum* (e.g. pathotype 2) infected potato wart material (max. 100 mg).

In addition to the external positive controls (PIC, PAC1 and PAC2), an internal positive isolation control is used to monitor each individual sample separately (specific amplification of plant COX gene). The use of this internal control is not necessary in case of *S. endobioticum* detection in winter spore suspension.

- 1. Interpretation of results

The cycle cut off value for both *S. endobioticum* P1, non-P1 and the Plant COX gene is set at 40, and was obtained using the equipment, materials and chemistry used as described in this appendix.

The cycle cut off value needs to be verified in each laboratory when implementing the test for the first time. To assign results from real-time PCR-based tests the following criteria should be followed:

*Adjusting threshold settings*

- Use PAC1 and PAC2 *S. endobioticum* 1(D1) and non-1(D1) to determine the threshold setting for both probes for each real-time PCR run. Non-P1 strains give a false positive VIC signal without producing exponential amplification curves. Increase the VIC threshold setting so that the false positive non-1(D1) signal does not exceed the threshold.

*Verification of the controls*

- NAC should be negative (Ct > cut off) for both *S. endobioticum* 1(D1), non-1(D1) and the Plant COX gene.
- NIC should be negative (Ct > cut off) for both *S. endobioticum* 1(D1), non-1(D1) 1 and produce an exponential amplification curve, and a Ct value below the cut off value for the Plant COX gene.
- PAC1 and PAC2 *S. endobioticum* 1(D1), should produce an amplification curve, and a Ct value below the cut off value for both *S. endobioticum* P1 (VIC) and the Plant COX gene. Please note that 1(D1 strains can give a false positive FAM signal (non-1(D1) probe).
- PAC1 and PAC2 *S. endobioticum* non-P1 should produce an exponential amplification curve, and a Ct value below the cut off value for both *S. endobioticum* non-P1 (FAM) and the Plant COX gene. The VIC signal should be negative (Ct > cut off and/or no exponential amplification curve)
- PIC should produce an exponential amplification curve, and a Ct value below the cut off value according to the pathotype used.

*When these conditions are met:*

- A test will be considered positive for *S. endobioticum* P1 if it produces an amplification curve, and a Ct value below the cut off value for *S. endobioticum* 1(D1) (VIC) . Please note that 1(D1) strains can give a false positive FAM signal.
- A test will be considered positive for *S. endobioticum* non-1(D1) if it produces an amplification curve, and a Ct value below the cut off value for *S. endobioticum* non-1(D1) (FAM).
- A test will be considered negative, if it produces no exponential amplification curve and/or a Ct value equal or above the cut off value for *S. endobioticum* 1(D1), non-1(D1), and for the Plant COX gene an exponential amplification curve, and a Ct value below the cut off value.
- Tests should be repeated if any contradictory or unclear results are obtained.

**SI table 1.** Homogeneity results TPS samples rounds 1 (wart material) and 2 (resting spores). Qualitative test results are provided for the Sendo PCR, whereas mean Ct values and standard deviations are provided for the Sendo TaqMan, 1(D1) TaqMan (both 1(D1) and non-1(D1) test) and COX TaqMan.

|  | Sendo PCR | Sendo TaqMan | 1(D1) TaqMan | non-1(D1)  TaqMan | COX  TaqMan |
| --- | --- | --- | --- | --- | --- |
| TPS round 1 – wart material | | | | | |
| 1(D1) | + | 16.3 (1.0) | 25.9 (1.6) | 26.8 (2.0) | 19.1 (1.6) |
| 2 (G1) | + | 19.6 (2.2) | - | 28.4 (2.2) | 19.9 (1.6) |
| 6(O1) | + | 15.5 (1.2) | - | 24.6 (1.1) | 18.3 (0.9) |
| 18(T1) | + | 18.1 (1.7) | - | 26.9 (1.6) | 19.5 (0.9) |
| 38(Nevsehir) | + | 18.7 (1.3) | - | 27.5 (1.4) | 20.4 (0.9) |
| Healthy potato | - | - | - | - | 19.9 (0.6) |
| TPS round 2 – resting spores | | | | | |
| 1(D1) 5000 sps^a^ | + ^b^ | 30.2 (0.5) | 37.6 (0.9) ^b^ | 37.5 (0.7) ^b^ | N.A.^c^ |
| 500 sps | + ^b^ | 33.8 (0.4) | - | - | N.A. |
| 50 sps | - | 36.2 (0.5)^b^ | - | - | N.A. |
| 5 sps | - | 38.4 ^b^ | - | - | N.A. |
| 6(O1) 5000 sps | + ^b^ | 30.3 (0.5) | - | 35.8 (0.6) | N.A. |
| 500 sps | + ^b^ | 33.4 (1) | - | 38.0 ^b^ | N.A. |
| 50 sps | - | 36.3 (0.3) ^b^ | - | - | N.A. |
| 5 sps | - | 38.9 ^b^ | - | - | N.A. |
| MGW | - | - | - | - | - |

a. resting spores per sample (10 µL molecular grade water), b. sample below the limit of detection, and with repeatability scores <100%, c. not applicable

**SI table 2.** Average repeatability per test matrix combination determined using TPS results. The number of samples analysed is shown in brackets. The number of samples included in the analysis for round 2 is less than those included in round 1 because less biological duplicates were provided to the TPS partners. In addition, removing datasets from the analysis with incorrect results obtained for the control samples occurred more frequently in round 2.

| TPS round | Sendo PCR |  | Sendo TaqMan |  | 1(D1) and non-1(D1) TaqMan |
| --- | --- | --- | --- | --- | --- |
| 1 (wart material) | 94% (52) |  | 94% (52) |  | 98% (52) |
| 2 (resting spores) | 64% (28) |  | 83% (24) |  | 29% (14) |

**SI table 3.** Robustness – disruption methods for wart material. Qualitative and quantitative results provided by TPS partners

| Qualitative |  | Sendo PCR | | | Sendo TaqMan | | | 1(D1) and non-1(D1) TaqMan | | |
| --- | --- | --- | --- | --- | --- | --- | --- | --- | --- | --- |
| sample | disruption method | samples tested | PA^a^ | *p* | samples tested | PA | *p* | samples tested | PA | *p* |
| wart 1(D1) | manual | 6 | 1.00 | 0.603 | 5 | 1.00 | 0.642 | 6 | 1.00 | 0.271 |
|  | Mechanical | 23 | 0.96 |  | 24 | 0.96 |  | 22 | 0.86 |  |
| healthy potato | manual | 6 | 1.00 | 1.000 | 6 | 0.67 | 0.185 | 6 | 1.00 | 1.000 |
|  | Mechanical | 26 | 1.00 |  | 26 | 0.50 |  | 26 | 1.00 |  |
| Quantitative |  | Sendo TaqMan | | | 1(D1) TaqMan | | | non-1(D1) TaqMan | | |
| sample | disruption method | samples tested | mean Ct | *p-*value | samples tested | mean Ct | *p-*value | samples tested | mean Ct | *p-*value |
| wart 1(D1) | manual | 6 | 20.1 | 0.002 | 6 | 30.7 | 0.162 | 6 | 27.6 | 0.515 |
|  | Mechanical | 22 | 17.0 |  | 23 | 27.3 |  | 23 | 26.7 |  |

a. Positive agreement

**SI table 4.** Intralaboratory analytical sensitivity results for the three different tests using wart material and winter spore suspensions

|  |  | Sendo PCR | |  | Sendo TaqMan | |  | 1(D1) samples | | |  | non-1(D1) samples | | |
| --- | --- | --- | --- | --- | --- | --- | --- | --- | --- | --- | --- | --- | --- | --- |
| Sample material |  | PA  (samples) | Amplicon |  | PA  (samples) | mean Ct (StDev) |  | PA  (samples) | mean Ct (StDev) | |  | PA  (samples) | mean Ct (StDev) | |
|  |  |  |  |  |  |  |  |  | 1(D1) TaqMan | non-1(D1) TaqMan |  |  | 1(D1) TaqMan | non-1(D1) TaqMan |
| Potato wart material | | | | | | | | | | | | | | |
| 100%^a^ |  | 100% (7) | + |  | 100% (7) | 20.3 (2.0) |  | 100% (3) | 28.3 (1.7) | 29.4 (2.4) |  | 100% (4) | - | 29.5 (1.1) |
| 10% |  | 100% (7) | + |  | 100% (7) | 23.7 (1.9) |  | 100% (3) | 31.8 (1.6) | 32.8 (2.3) |  | 100% (4) | - | 32.9 (1.1) |
| 1% |  | 100% (7) | + |  | 100% (7) | 27.1 (2.0) |  | 100% (3) | 35.0 (2.2) | 36.3 (3.2) |  | 100% (4) | - | 37.0 (2.2) |
| 1·10^-1^ % |  | 57% (7) | w^c^ |  | 43% (7), *100%(7)*^d^ | 30.4 (1.9) |  | 33% (3) | 37.7 | 37.0 |  | 50% (4) | - | 37.4 (0.3) |
| 1·10^-2^ % |  | 14% (7) | w |  | 0 (7), *100%(7* | 34.0 (2.3) |  | 33% (3) | 37.5 | 39.5 |  | 0 (4) | - | - |
| 1·10^-3^ % |  | 0 (7) | - |  | 0 (7),  *86% (7)* | 37.0 (2.0) |  | 0 (3) | - | - |  | 0 (4) | - | - |
| 1·10^-4^ % |  | 0 (7) | - |  | 0 (7),  *29% (7)* | 37.5 (1.4) |  | 0 (3) | - | - |  | 0 (4) | - | - |
| 1·10^-5^ % |  | 0 (7) | - |  | 0 (7),  *14%(7)* | 38.6 |  | 0 (3) | - | - |  | 0 (4) | - | - |
| 1·10^-6^ % |  | 0 (7) | - |  | 0 (7) | - |  | 0 (3) | - | - |  | 0 (4) | - | - |

**SI table 4 (continued).** Intralaboratory analytical sensitivity results for the three different tests using wart material and winter spore suspensions

|  |  | Sendo PCR | |  | Sendo TaqMan | |  | 1(D1) samples | | |  | non-1(D1) samples | | |
| --- | --- | --- | --- | --- | --- | --- | --- | --- | --- | --- | --- | --- | --- | --- |
| Sample material |  | PA  (samples) | Amplicon |  | PA  (samples) | mean Ct (StDev) |  | PA  (samples) | mean Ct (StDev) | |  | PA  (samples) | mean Ct (StDev) | |
|  |  |  |  |  |  |  |  |  | 1(D1) TaqMan | non-1(D1) TaqMan |  |  | 1(D1) TaqMan | non-1(D1) TaqMan |

| Resting spore suspensions | | | | | | | | | | | | | | |
| --- | --- | --- | --- | --- | --- | --- | --- | --- | --- | --- | --- | --- | --- | --- |
| 5000^b^ |  | 82% (11) | + |  | 100% (11) | 29.8 (0.6) |  | 40% (6) | 37.6 (0.9) | 37.5 (0.7) |  | 100% (5) | - | 35.8 (0.6) |
| 500 |  | 80% (10) | w |  | 100% (10) | 33.5 (0.8) |  | 0 (5) | - | - |  | 20% (5) | - | 38.0 |
| 50 |  | 40% (10) | w |  | 90% (10) | 35.8 (0.5) |  | 0 (5) | - | - |  | 0 (5) | - | - |
| 5 |  | 0 (10) | - |  | 40% (10) | 38.2 (0.6) |  | 0 (5) | - | - |  | 0 (5) | - | - |

a. relative infection rate: undiluted naturally infected wart material is regarded as 100% infected, b. resting spores per sample (10 µL molecular grade water), c. weak positive amplicon, d. positive agreement based on a Ct cut-off value of 30, and 40 (italics)

**SI table 5.** Intralaboratory analytical specificity using warted potato tissue

| Strain | Pathotype | Origin | Sendo PCR | Sendo TaqMan | 1(D1) TaqMan | | non-1(D1) TaqMan | COI |
| --- | --- | --- | --- | --- | --- | --- | --- | --- |
| MB42 | 1(D1) | Netherlands | + | 17.4 | 26.0 | 27.8 | | 18.5 |
| MB69 | 1(D1) | Sweden | + | 21.8 | - | 31.4 | | 21.5 |
| 5022364 | 1(D1) | Netherlands | + | 15.2 | 23.1 | 26.6 | | 18.7 |
| MB81 | 1(D1) | Ireland | + | 19.9 | 32.1 | 29.0 | | 21.1 |
| MB08 | 2(G1) | Netherlands | + | 20.1 | - | 28.4 | | 18.1 |
| MB10 | 6(O1) | Netherlands | + | 17.6 | - | 26.6 | | 18.4 |
| MB14 | 18(T1) | Germany | + | 20.1 | - | 28.5 | | 21.0 |
| MB55 | 18(T1) | Sweden | + | 21.5 | - | 29.9 | | 21.2 |
| 4112001 | 18(T1) | Germany | + | 28.1 | - | 36.9 | | 29.8 |
| 39.9.2893 | 18(T1) | Sweden | + | 22.1 | - | 30.7 | | 21.4 |
| MB85 | 18(T1) | Greece | + | 20.5 | - | 29.5 | | 21.0 |
| MB82 | 18(T1) | Germany | + | 16.9 | - | 26.2 | | 22.8 |
| MB15 | 18(T1) | Germany | + | 18.3 | - | 27.1 | | 19.4 |
| MB86 | 18(T1) | Greece | + | 24.6 | - | 32.9 | | 19.8 |
| MB56 | 38(Nevsehir) | Turkey | + | 18.5 | - | 27.1 | | 20.2 |
